# How Do Undiagnosed Symptoms of Maternal Psychological Distress During the Postnatal Period Affect Child Developmental Outcomes?

**DOI:** 10.1007/s10995-019-02749-w

**Published:** 2019-06-21

**Authors:** Kerry Bell, Karen Bloor, Catherine Hewitt

**Affiliations:** 1 York Trials Unit, Department of Health Sciences, University of York, York, YO10 5DD UK; 2 Department of Health Sciences, University of York, York, YO10 5DD UK

**Keywords:** Maternal psychological distress, Child development, Behaviour problems, Multilevel modelling, Cognitive development

## Abstract

**Objectives:**

Evidence suggests that maternal psychological distress is an under-diagnosed condition that can have lasting impacts on child outcomes. Models based solely on maternal outcomes have not found screening to be cost-effective. This research explores the effects of self-reported maternal psychological distress on children’s language and behavioural development up to the age of 7.

**Methods:**

Using longitudinal survey data from 10,893 families in the UK Millennium Cohort Study, multilevel models are used to explore the differential effects of maternal diagnosed and treated depression versus untreated maternal psychological distress during the postnatal year on longer-term child outcomes.

**Results:**

Both diagnosed and treated depression and self-reported maternal psychological distress have detrimental effects on child behavioural development. Behavioural outcomes up to age 5 were better for children of women who received treatment for depression, compared with children those whose mothers’ psychological distress was untreated, but this was not maintained to age 7. Little or no evidence of a difference was found between maternal psychological distress and child language development.

**Conclusions for Practice:**

This research highlights the lack of effectiveness of existing treatment for maternal psychological distress both to benefit child development and to provide long-term symptom remediation for women. Future research could aim to identify more effective treatments for both women and children.

## Significance

*What is already known on this subject?* Maternal psychological distress is an under-diagnosed condition that can have lasting impacts on child outcomes. Screening all women for symptoms of distress could aid in case finding and subsequent intervention but it is not currently considered costeffective in the UK.

*What this study adds?* Symptoms of maternal distress 9 months postpartum are associated with long-term child behavioural problems evident to the age of 7. Receipt of treatment for symptoms does not appear to improve child outcomes however the data set is limited in its capacity to distinguish between different treatment effects. Future research into the effects of interventions should also consider child outcomes.

## Introduction

Maternal postnatal depression is a major public health issue because of its globally high prevalence amongst women in both developed and developing countries (Halbreich and Karkun 2006). The most widely used estimate of prevalence for postnatal depression in the UK suggests that 14% of all postnatal women are affected (OHara and Swain 1996). Considering that in the UK more than 700,000 women give birth each year (Office for National Statistics 2013) this estimate would suggest that around 98,000 women will fall into this category annually.

Although both effective and cost-effective treatments exist to relieve the symptoms of postnatal depression (Hendrick 2003; McCrone et al. 2004), evidence suggests that it is an under-diagnosed condition (Hearn et al. 1998). One method of increasing detection rates would be to screen all postnatal women using an established screening tool. NICE currently recommend the use of two case finding questions to aid in case detection, known as the Whooley questions (Whooley et al. 1997) which ask; “during the past month, have you often been bothered by feeling down, depressed or hopeless?” and “during the past month, have you often been bothered by little interest or pleasure in doing things?” An affirmative response to one or both of these questions warrants further evaluation. Formal universal screening with a more structured case finding measure such as the Edinburgh Postnatal Depression Scale (Cox et al. 1987) is not currently viewed as cost effective based on maternal outcomes alone, a decision driven by high costs associated with false positive results (Hewitt et al. 2009).

In addition to affecting the women themselves, maternal depression may affect the quality and style of parenting (Broth et al. 2004) and can lead to a less secure mother–child attachment (Martins and Gaffan 2000). Depressed mothers tend to report feeling less attached to and more negative towards their children (Nagata et al. 2003). Alterations in parenting behaviour can have an adverse impact on the psychosocial and neurobiological development in children (Newport et al. 2002). Systematic reviews have reported marked effects of maternal depression on behavioural development (Grace et al. 2003; Goodman et al. 2011) whilst the relationship with cognitive development appears weaker. A review carried out in 2003 reported mixed results in terms of the relationship between maternal postnatal depression and child cognitive outcomes (Grace et al. 2003). Only two identified studies reported significant effects on cognitive assessments (Murray 1992; Hay and Kumar 1995) whilst the remaining five showed no significant effects. Another more recent study noted a correlation between the duration of maternal depression and language development suggesting that we may only see an effect when depression is more persistent (Quevedo et al. 2012).

Even though the association between maternal depression and child development is well established, it is unknown whether self-reported symptoms of psychological distress have a similar effect on children’s development to formally diagnosed depression. If children are affected by symptoms of psychological distress (that may or may not meet the threshold for clinical significance), current evidence may underestimate the effectiveness and cost-effectiveness of screening for and treating such symptoms that may not be identified by the Whooley questions. This analysis aims to assess how both diagnosed and treated depression and self-reported symptoms of psychological distress affect child language and behavioural development up to the age of 7.

Given the range and breadth of factors that can influence developmental trajectories in children, it is crucial to consider any covariates that may act to either reduce or accentuate the effect of maternal depression on child development. For instance, Kiernan and Huerta (2008) found that economic deprivation and maternal depression both affect the cognitive and emotional well-being of children with children faring worse when both are present. Conversely, children born into higher earning families may be offered some protection against the detrimental effects of maternal depression as parents are able to invest in resources that benefit development. Other factors which may moderate the impact of maternal depression include household structure (i.e. coming from a dual parent household) (Goodman et al. 2011) and maternal education (Grace et al. 2003). Shonkoff and Phillips present an overview of the important contexts for child development encompassing both family and child level characteristics (Shonkoff and Phillips 2000). Building on this work we conducted a comprehensive review of the literature to identify the key factors that can impact on child development. We identified a range of important variables at the household level such as maternal qualifications (Poobalan et al. 2007), maternal ethnicity (Goodman et al. 2011), socioeconomic disadvantage (indicated using median income data) (Goodman et al. 2011; Peacock et al. 2013), maternal age (Peacock et al. 2013), number of previous live births (Chaimay et al. 2006), household structure (Chaimay et al. 2006; Goodman et al. 2011) and maternal employment (Poobalan et al. 2007) in addition to a number of child characteristics including age (Goodman et al. 2011; Breinholst et al. 2012), gender and ethnicity (Goodman et al. 2011). These factors were included as covariates in the analysis to help isolate the independent effect of maternal mental health status.

## Methods

### Sample

This study uses data from the Millennium Cohort Study (MCS) to compare three sets of children:

1. Children of women with diagnosed depression who are receiving treatment
2. Children of women who show signs of psychological distress based on self-report assessments but who are not receiving any treatment
3. Children of women who are not being treated for depression and appear to have few or no symptoms of psychological distress 9 months after birth.

The analytical sample comprises 10,893 mother–child pairs drawn from the MCS in 2000/01, followed for 7 years. The MCS is a large-scale longitudinal survey of 19, 517 infants born in the four constituent countries of the United Kingdom, designed to oversample those living in disadvantaged circumstances (Plewis 2004). As the MCS specifically targeted ethnic minorities these are reasonably well represented in the data with 11.5% of the cohort being non-white. Families from low-income households are also well represented as almost a third of the sample population is classified as being in relative poverty (with household disposable income lower than 60% of median income levels). The MCS included women across a broad age range (13–51 years). In addition to baseline data at birth, follow-up data was also available for the families: 3, 5 and 7 years after birth at the time of analysis.

The sampling method for the analysis was based on pre-specified inclusion criteria, namely, the mother must have been the primary respondent remaining active in the study to provide data at each time point and the mother must have provided data on her mental health and well-being based on the Malaise Inventory (Rutter 1970; Grant et al. 1990) and study specific mental health questions. In families comprising multiple births, only the first cohort infant was included in the analysis to avoid repeatedly sampling the same mother.

**Fig. 1:**
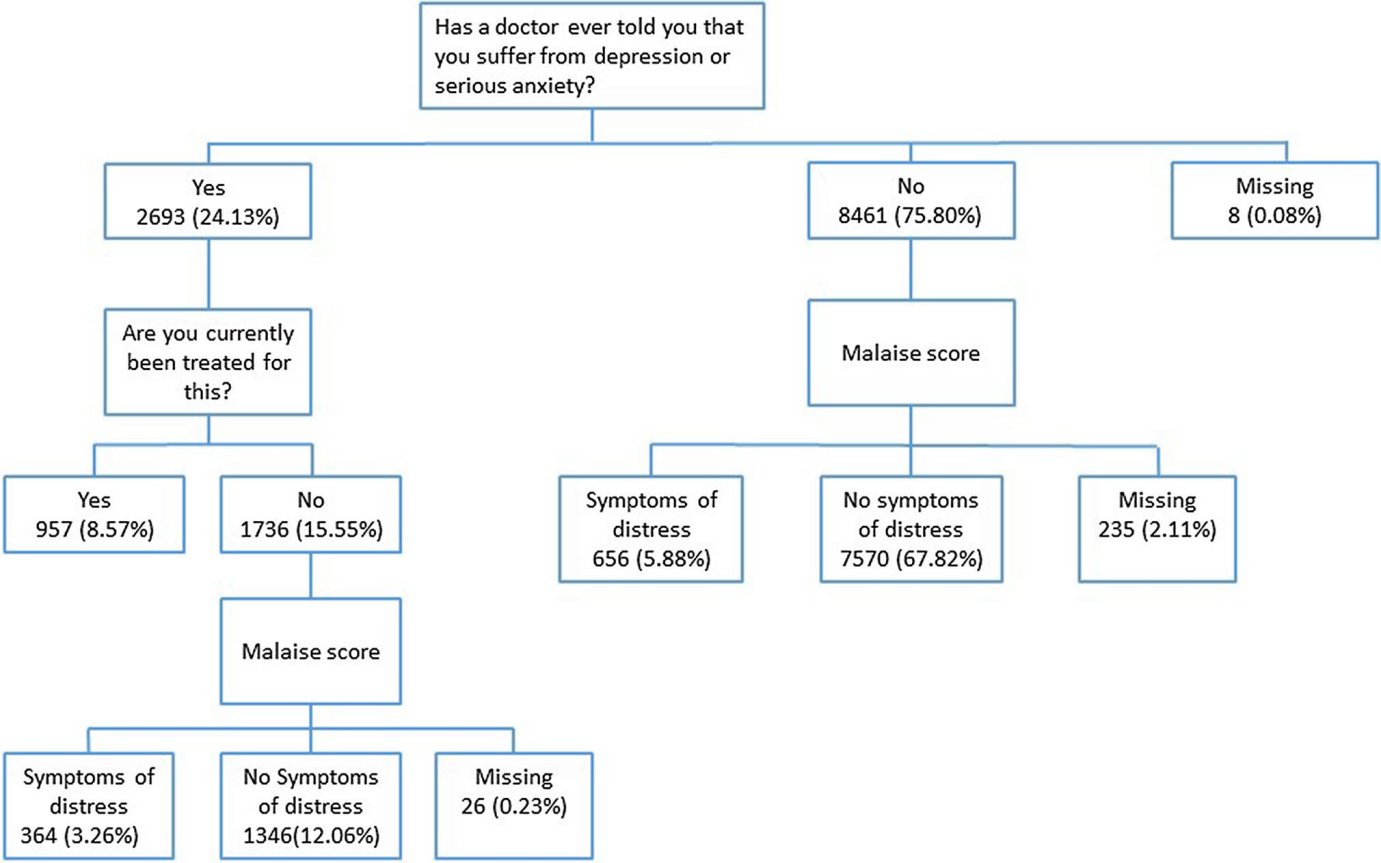
Flow chart demonstrating group derivation

Women’s mental health status was determined using two methodologies. Firstly, using the first sweep of questionnaire responses, women were categorised as having diagnosed and treated depression if they responded to two questions affirmatively, namely, *‘has a doctor ever told you that you suffer from depression or serious anxiety?’* and *‘are you currently being treated for this?’*. Secondly, women were categorised as having untreated symptoms of psychological distress if they did not meet the inclusion criteria for the former category, but did score above a recognised threshold of four points on the nine-item Malaise Inventory, which has been used in similar research contexts (Bartley 2004; Kiernan and Huerta 2008; Mensah and Kiernan 2010). Though the Malaise Inventory cannot confirm the presence of clinical-level depression, it provides an indication of symptoms, which can be indicative of underlying mental illness. Women who did not meet either of these conditions were considered neither depressed nor experiencing symptoms of psychological distress. The categorisation method is summarised in Fig. 1.

Based on this, 957 women were identified as receiving treatment for depression at the baseline survey, 1020 women were identified as having untreated symptoms of psychological distress at baseline and 8916 women were identified as having no current mental health or well-being issues at baseline.

### Child Outcome Measures

#### Cognitive Development

A number of components of the British Ability Scales (BAS) were included at each of the MCS follow up data sweeps, however these varied by sweep. Assessments of language were included at each sweep thus reflecting a usable repeated measure for the present analysis. Vocabulary skills were measured as an indicator of cognitive abilities specifically, the Naming Vocabulary subscale at ages 3 and 5 years and the Word Reading subscale at age 7 years (Elliott 1983). These scales are moderately correlated (r = 0.34) (Hanson et al. 2010), however, as they measure slightly different aspects of language, direct changes in scores cannot be mapped over time. Consequently, standardised scores adjusting for child age, norming sample mean and standard deviation were dichotomised according to the lowest scoring 10% of children. This allowed odds ratios to be calculated for children scoring in the lowest decile of the distribution of children.

#### Behavioural Development

Children’s behavioural development was assessed at ages 3, 5, and 7 years using the Strengths and Difficulties Questionnaire (SDQ) (Goodman 1997). This is a 25-item behavioural screening questionnaire addressing five different dimensions of behaviour: inattention/hyperactivity, conduct, peer relations, emotional development and prosocial behaviour. The SDQ has good reliability and validity and responses from parents are generally consistent with more independent respondents such as teachers (Mieloo et al. 2012). In the MCS, each attribute was rated by the mother using a scale from 0 to 2 (not true, somewhat true, and certainly true). Scores from the first four dimensions can be combined to generate a total difficulties score ranging from 0 to 40 where higher scores reflect increasingly marked behaviour problems. In the analysis presented in this paper these scores were analysed on both a continuous basis and by dichotomising children according to an established threshold of 14 points, indicating moderate to high levels of behaviour problems (Meltzer 2000; Goodman 2001; Goodman and Goodman 2009).

### Statistical Analysis

The analysis compared cognitive and behavioural outcomes of children according to maternal mental health status 9 months after birth. Multilevel models were fitted by outcome to the data, with time points nested within children to allow for clustering of data within each child. Children of women with untreated symptoms of psychological distress were compared with children of women with diagnosed and treated depression and both of these groups were compared to children of non-depressed women. Each model adjusted for the fixed effects of group (treated depression, untreated distress or non-depressed), time (3, 5, or 7 years after birth) and a set of pre-specified covariates. These covariates were based on an extensive review carried out prior to the analysis and included parental level (maternal qualifications, employment status, age, ethnicity, and number of previous live births), household level (socioeconomic disadvantage, single or dual parent household) and child level attributes (age, gender and ethnicity).

Appropriate covariance patterns were selected for the models based on fit as determined by Akaike’s Information Criteria (AIC) and log likelihood values. Estimates of differences in children’s language and behavioural development between the groups were statistically assessed at individual time points. Any response bias arising from families that did not provide outcome data at all three follow-up time points was minimised by using a multilevel model. The impact of missing data was assessed by a sensitivity analysis using Markov Chain Monte Carlo imputation (Schafer 1997). All data preparation was conducted in Stata 11 and all modelling was conducted using SAS 9.2. Sampling weights available in the dataset were used to adjust for the probability of the participants being included in the study, and attrition between the first and final data collection points.

### Ethical Approval

The Millennium Cohort Study was conducted in accordance with the ethical standards laid down in the 1964 Declaration of Helsinki and its later amendments. In full, the first sweep of the Millennium Cohort Study received ethics approval from National Health Service Ethical Authority in February 2001 (MREC/01/6/19), and the second and third received approval from the London Multi-Centre Research Ethics Committee in September 2004 (MREC/03/2/022) and December 2005 (05/MRE02/46). The fourth sweep of the Millennium Cohort Study received ethics approval from the Northern and Yorkshire Multi-Centre Research Ethics Committee of the NHS in February 2008 (07/MRE03/32). No further research ethics approval was required for present study.

## Results

The demographic profiles for the women and their children at the first data sweep are presented in Table 1. No major differences were identified between these demographic profiles, however, more women with untreated symptoms of psychological distress compared with diagnosed and treated depression reported being in poverty and being from an ethnic minority suggesting there may be some relationship between circumstances and mental health within this sample.

**Table 1:** Participant demographic characteristics according to maternal mental health group

	Diagnosed N = 957	Distressed N = 1020	Non-depressed N = 8916
Maternal characteristics			
Age at CM birth (years), mean	28.7	28	29.2
Qualification level			
Level 1	10.0	10.4	7.2
Level 2	34.5	28.5	29.1
Level 3	15.8	15.6	15.1
Level 4	21.7	23.3	32.8
Level 5	1.78	3.0	4.1
Overseas only	2.3	3.4	1.8
None	13.9	15.8	9.9
Lone parent	19	19.7	12.1
In poverty	37.8	41.8	25.5
First live birth	36.5	38.5	43
In employment	40.7	44.1	53
Ethnicity			
White	93.0	82.2	90.0
Mixed	0.4	0.9	0.7
Indian	1.5	4.6	1.9
Pakistani/Bangladeshi	3.0	7.7	3.7
Black/Black British	1.6	3.3	2.5
Other	0.5	1.5	1.1
Child characteristics			
Child age (months), mean	9.2	9.2	9.2
Male	48.8	52.8	50.4
Ethnicity			
White	91.4	81.2	88.7
Mixed	2.3	2.8	2.5
Indian	1.6	4.3	1.9
Pakistani/Bangladeshi	2.9	7.4	3.7
Black/Black British	1.6	3.3	2.4
Other	0.2	1.1	0.8

Values are unweighted percentages of the sample unless stated otherwise

Unadjusted mean scores on both the language and behavioural assessments are presented in Table 2. Few differences are apparent between the three maternal mental health groups in terms of mean language scores at each of the three follow-up age groups, however there is some evidence of a relationship with scoring in the lowest decile of children, particularly at ages 3 and 5. A more pronounced effect is evident for behavioural development with mean scores being the greatest for children of untreated distressed women. This is further reflected in the proportion of children scoring above threshold for behavioural problems.

**Table 2:** Mean developmental outcome scores and proportions of children scoring in the lowest performing decile by maternal mental health group

	N	Mean (SD)	% Scoring in lowest 10%
Child language development, (BAS: naming vocabulary)			
Age 3			
Non-depressed mother	8493	17.21 (4.51)	8.29
Diagnosed mother	913	16.88 (4.49)	9.20
Distressed mother	960	16.05 (5.90)	14.58
Age 5			
Non-depressed mother	8445	14.85 (3.18)	7.92
Diagnosed mother	901	14.42 (3.05)	9.10
Distressed mother	948	14.12 (3.18)	12.55
Child language development, (BAS: word reading)^a^			
Age 7			
Non-depressed mother	8282	45.50 (18.83)	9.65
Diagnosed mother	890	41.84 (19.63)	14.49
Distressed mother	928	42.31 (20.09)	14.44
Child behavioural development, (SDQ)^b^			
Age 3			
Non-depressed mother	6179	8.37 (4.67)	10.45
Diagnosed mother	620	10.12 (5.51)	20.00
Distressed mother	593	11.51 (5.55)	28.84
Age 5			
Non-depressed mother	7162	6.25 (4.30)	4.80
Diagnosed mother	721	8.30 (5.40)	13.04
Distressed mother	727	9.30 (5.50)	17.88
Age 7			
Non-depressed mother	7445	6.49 (4.83)	7.20
Diagnosed mother	767	9.03 (6.14)	18.25
Distressed mother	754	9.40 (6.15)	19.36

^a^Child language development was measured using the Naming Vocabulary or Word reading subscales of the British Ability Scales (BAS)

^b^Child behaviour development was measured using the Strengths and Difficulties Questionnaire (SDQ)

**Fig. 2:**
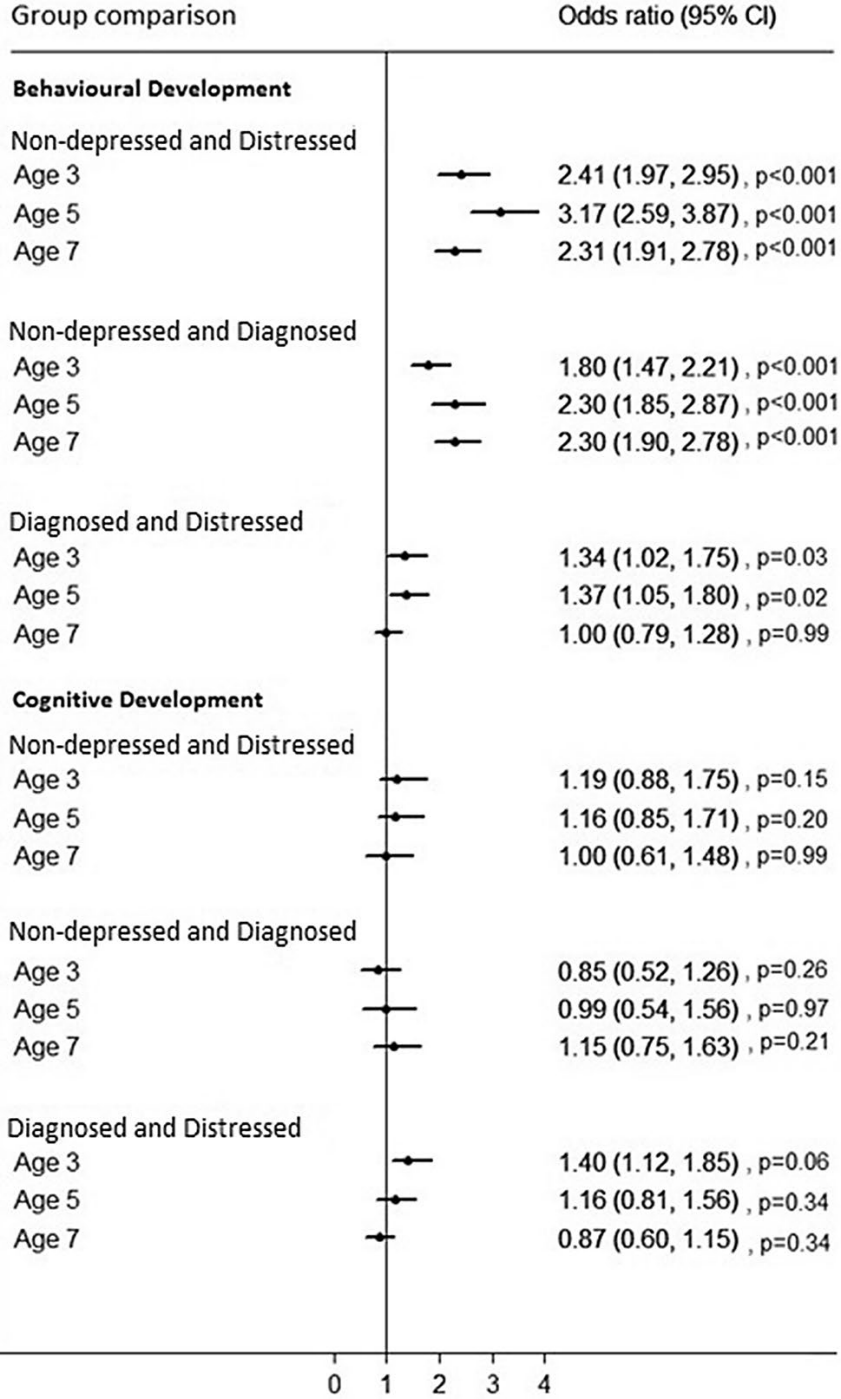
Multilevel analysis showing the association between maternal mental health and children’s language and behavioural outcomes over time

A multilevel model with compound symmetry covariance structure was found to be the most appropriate model for both the language and the behaviour assessments. There was little or no evidence of a difference in child language development between children of diagnosed and treated women and children of women showing signs of untreated psychological distress for most of the comparisons (Fig. 2). There was some evidence of a difference in language development problems at a single time point (age 3) with the odds being higher for those children of women who were categorised as showing signs of untreated psychological distress compared to those who were diagnosed and receiving treatment for depression (OR 1.40, 95% CI 1.12 to 1.85, p = 0.06).

Children’s behavioural development was modelled based on both the total SDQ score as a continuous measure and dichotomised according to a known threshold. For the continuous SDQ scores, children of women who were receiving treatment for depression or showed signs of psychological distress at 9 months after birth performed consistently significantly worse than children of women who had no symptoms (Table 3).

**Table 3:** Multilevel analysis showing the association between maternal depression and children’s SDQ total difficulties scores over time

Child behavioural development, (SDQ)	Coef	95% CI	p
Non-depressed and distressed			
Age 3	0.26	0.19–0.33	<.001
Age 5	0.37	0.31–0.43	<.001
Age 7	0.31	0.25–0.37	<.001
Non-depressed and diagnosed			
Age 3	0.14	0.08–0.20	<.001
Age 5	0.24	0.18–0.30	<.001
Age 7	0.27	0.22–0.33	<.001
Diagnosed and distressed			
Age 3	0.12	0.03–0.19	0.006
Age 5	0.1	0.04–0.18	0.002
Age 7	0.03	− 0.03 to 0.1	0.338

Focusing on the dichotomous SDQ scores, the odds of children having behavioural difficulties were higher for both children of women who were receiving treatment for depression and of women who were showing signs of psychological distress at 9 months after birth compared to children of women who had no symptoms, at all three time points (Fig. 2). Smaller differences were observed in the comparison between children of women being treated for depression and children of women with untreated symptoms of psychological distress. At age 3, the odds of children having behavioural difficulties were higher for children of women with untreated symptoms of psychological distress compared with women who were receiving treatment for depression (OR 1.34, 95% CI 1.02 to 1.75), the odds ratio rising slightly to 1.37 (95% CI 1.05 to 1.80) at age 5. At age 7 there was no longer a significant increased odds of a child from a mother with untreated symptoms of psychological distress scoring above threshold for behavioural difficulties compared with a child from a mother receiving treatment (OR 1.00, 95% 0.61 to 1.48).

### Interactions

No variations by gender were found for any of the outcomes explored: cognitive development model (p-interaction = 0.31), the behavioural model (p-interaction = 0.42). Similarly for ethnic differences (cognitive development p = 0.15 and total difficulties p = 0.60).

## Discussion

### Principal Findings

This analysis demonstrates that there are clear associations between maternal mental health during the first postnatal year and children’s behavioural development through to school-age. These findings are consistent with similar work in establishing the association between maternal depression and early child outcomes using the MCS (Kiernan and Huerta 2008; Kiernan and Mensah 2009a, b; Mensah and Kiernan 2010), but this analysis extends the follow-up of children to age 7 allowing for a more longitudinal picture of developmental changes.

More importantly, these findings highlight the importance of untreated and potentially undiagnosed symptoms of maternal psychological distress, and the detrimental effect this can have on children’s development, which appears equal to depression that has been clinically diagnosed and treated. Treatments for maternal depression during the postnatal year at the time of data collection (2000/01) apparently did not have sustained long-term benefits for children, improving outcomes only up to age 5 but not to age 7.

Although studies have explored the effects of depression on children based on self-report or clinical diagnosis individually (Grace et al. 2003; Goodman et al. 2011), this is the first to assess the distinct associations of diagnosed and treated depression and untreated and potentially undiagnosed symptoms of psychological distress on children’s longitudinal development, both in terms of language and behaviour, within the same sample.

This analysis adds to the discussion of whether maternal depression symptoms effect cognitive development, a currently disputed area. Consistent with other work conducted within the MCS (Kiernan and Mensah 2009a, b) we found no significant association with language development, reflective of cognition, for either of the groups.

### Limitations

The present works makes use of cohort study data hence is constrained by the variables available in the dataset. This presents several limitations. Firstly, the nine-item Malaise Inventory is not a diagnostic instrument thus can only give us an indication of symptoms of psychological distress rather than a formal diagnosis of depression. Although the full 24-item version of the inventory has good reliability and validity, the nine-item version has not been validated against more diagnostic instruments such as the EPDS (Grant et al. 1990). The Malaise Inventory is not a condition specific instrument hence there may be overlap with other psychological conditions such as psychotic illness. However, this instrument has been used comparable work (Bartley 2004; Kiernan and Huerta 2008; Mensah and Kiernan 2010) and provides a good indicator that women are experiencing symptoms commonly associated with depression. Secondly, the categorisation method is unable to separate those women who may have been diagnosed with depression but who had refused or were awaiting treatment, therefore there may be some contamination of individuals between the different groups. Finally, the analysis is unable to account for the time of symptom onset as no mental health history was taken from the women, meaning that the depression observed during the postnatal period may not have a specific postnatal onset and may be part of a chronic condition.

The study is somewhat limited by dependence on measures that rely on mothers reporting on their children, particularly the primary outcome measure, child behaviour. Mothers in general are not impartial observers of their children’s behaviour and there is some evidence that mothers with depression may be particularly prone to viewing their children’s behaviour more negatively (Renouf and Kovacs 1994). However, others have argued that mothers with depression may actually be more sensitive to children’s behaviour and may in fact make more accurate reflections than those women without depression (Richters 1992; Glascoe 2005).

Previous research has highlighted a cumulative effect of exposure to symptoms of maternal depression whereby increased duration of depression has a greater effect on developmental outcomes (Ashman et al. 2008; Kiernan and Mensah 2009a, b). Given that maternal distress was measured at only one time point (9 months after birth), we cannot account for the effect of chronic or concurrent depression at any of the follow-up time points. However, the aim of this research was to explore whether screening during the postnatal period, a critical period for development, can have lasting benefits irrespective of future exposure to distress or indeed any of the predictive factors of development. This was found not to be the case as the benefit of treatment was not sustained.

Finally, the analysis was restricted to those receiving any treatment, as we had no information on the type of treatment received. Consequently, the analysis presented in this paper is unable to account for the differential effects of different treatment types.

### Implications and Further Research

Language is only one aspect of cognitive ability. Although no significant association was found between treated maternal depression or untreated maternal distress during the postnatal year and subsequent language development, we cannot take this to mean that there is no effect on other aspects of cognition. More research is required to fully understand the consequences of maternal depression on all aspects of child cognition.

These results demonstrate clearly that symptoms of psychological distress are sufficient to have long lasting detrimental effects on children’s behavioural development. Improving strategies to diagnose symptoms of depression may improve detection rates of these symptoms. However, before further efforts are made to identify symptoms of depression, treatments for postnatal depression need to be developed and refined to ensure not only that the maternal symptoms are being treated but also the effects of the women’ depressive symptoms on the children’s development are minimised.

Future research could focus on examining the differential effects of different treatments for maternal depression in terms of outcomes for children. This could provide important considerations for policy and treatment recommendations. Consideration should also be given to the effects of maternal mood over time for child outcomes, as research has indicated that there may be cumulative effects of exposure to depression (Brennan et al. 2000; Ashman et al. 2008; Kiernan and Mensah 2009a, b). The way in which treatment effects dissipated by age 7 may be indicative of systematic differences in the long-term mood of women who were receiving treatment, perhaps contributing to why these women were identified by clinicians.

The present study addresses the value of treating symptoms of maternal depression during the postnatal period. However, for many women depression and/or anxiety may emerge during pregnancy and continue into the postnatal period. Both pregnancy and the first year following birth present a unique opportunity for health care professionals to identify cases of depression due to increased contact with women. Future research could explore the potential long-term benefits of screening more formally for signs of antenatal depression as well as postnatal depression.

## Conclusion

Both children of women who are receiving treatment for depression and children of women with untreated and potentially undiagnosed symptoms of psychological distress are at greater risk of developing behavioural problems compared with children of women without issues. Diagnosing and effectively treating post-natal depression appears to have positive effects on child outcomes up to age 5, but these are not sustained to age 7. Treatments that improve maternal symptoms, such as cognitive behavioural therapy and counselling therapies, could incorporate measures to help prevent the development of behaviour problems in children, perhaps through more family based interventions or education in parenting skills. More research is required to investigate which treatments have the best outcomes for both women and children, and to establish what the effects of maternal mental health trajectories over time are.
